# Simulation and Analysis of Mechanical Properties of Silica Aerogels: From Rationalization to Prediction

**DOI:** 10.3390/ma11020214

**Published:** 2018-01-30

**Authors:** Hao Ma, Xiaoyang Zheng, Xuan Luo, Yong Yi, Fan Yang

**Affiliations:** 1 School of Materials Science and Engineering, Southwest University of Science and Technology, Mianyang 621010, China; mahao881208@gmail.com (H.M.); xyzheng1995@gmail.com (X.Z.); 2 Research Center of Laser Fusion, China Academy of Engineering Physics, Mianyang 621900, China; yangf711@mail.ustc.edu.cn

**Keywords:** silica aerogel, mechanical properties, simulation, finite volume method (FVM)

## Abstract

Silica aerogels are highly porous 3D nanostructures and have exhibited excellent physio-chemical properties. Although silica aerogels have broad potential in many fields, the poor mechanical properties greatly limit further applications. In this study, we have applied the finite volume method (FVM) method to calculate the mechanical properties of silica aerogels with different geometric properties such as particle size, pore size, ligament diameter, etc. The FVM simulation results show that a power law correlation existing between relative density and mechanical properties (elastic modulus and yield stress) of silica aerogels, which are consistent with experimental and literature studies. In addition, depending on the relative densities, different strategies are proposed in order to synthesize silica aerogels with better mechanical performance by adjusting the distribution of pore size and ligament diameter of aerogels. Finally, the results suggest that it is possible to synthesize silica aerogels with ultra-low density as well as high strength and stiffness as long as the textural features are well controlled. It is believed that the FVM simulation methodology could be a valuable tool to study mechanical performance of silica aerogel based materials in the future.

## 1. Introduction

Silica aerogels have attracted considerable attention due to the high porosity, low thermal conductivity, high surface area, and good chemical stability, etc. [1,2,3,4]. Because of these unique physio-chemical properties, silica aerogels have promising applications in many areas such as building insulation, structural materials, automotive and aerospace [5,6,7]. Despite this great potential, poor mechanical performance still limits its large-scale and commercial applications. Experimental studies have recently shown that the mechanical reliability of silica aerogels can be greatly reinforced by adding other coexisting materials into the aerogel such as polymer, carbon nanotubes, titanium, ceramic fiber, etc. [8,9,10]. However, the structure–property relationships of silica aerogels are still unclear due to its complex disorderly structures and limited experimental tools [11]. It is believed that computer simulation may be a feasible approach to investigate the relationships between mechanical performance and microstructures of silica aerogels.

The molecular dynamic (MD) method has been utilized to model the nanoporous structure and mechanical behavior of silica aerogels [12,13,14,15]. Although the MD method can reasonably predict the density-elastic modulus relationship of silica aerogels, it fails to simulate the compressive strength of the materials [5]. In addition, the length and time scale are much shorter in MD simulations, which also limits the application of MD method to investigate mechanical properties of silica aerogels on a large scale [16]. The finite element method (FEM) is a common numerical method that has been employed to model the mechanical behavior of aerogels at the microscopic scale [11,17,18,19]. Ma et al. [11] have generated a computational model of aerogels based on DLCA (diffusion-limited cluster aggregation) algorithm, and they use the FEM method and beam theory to successfully predict the power-law relationship between the elastic modulus and relative density of silica aerogels. However, in the FEM models, the material parameters do not represent the true ones of the silica aerogel such that it still remains challenging to directly apply FEM to evaluate the mechanical properties of silica aerogels. In this study, the simulation of mechanical performance of silica aerogels is carried out by the finite volume method (FVM), which is another numerical method to solve the complex engineering problems by discretizing the computational domains into finite volumes and the governing equations for each volume are then solved [20,21]. Compared to the molecular dynamic simulation and finite element method, FVM does not require a large number of computational resources and the implementation of FVM is rather convenient. In addition, the FVM method can represent the actual sample size, morphology and other sample states. Therefore, FVM has been demonstrated as a powerful technique to simulate the mechanical structures both in microscopic and macroscopic scales [20,21,22,23].

**Figure 1:**
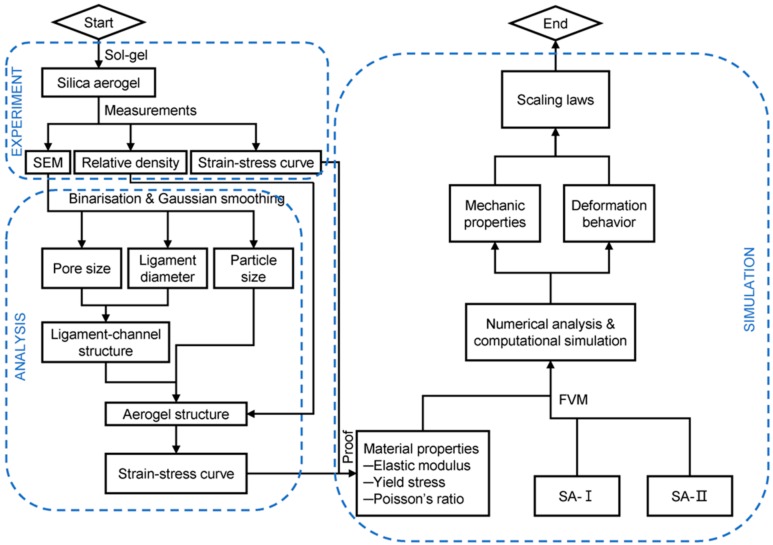
Flow chart for the experiment, modeling and simulation in this study.

Figure 1 presents the detailed experimental, modeling and simulation procedures in this study. Firstly, the silica aerogels with different geometric properties are fabricated by the sol-gel method. Based on the characterization results from the scanning electron microscope (SEM) and Brunauer–Emmett-Teller (BET) analysis, the geometrical properties of silica aerogel such as pore size, ligament diameter, and particle size can be obtained. According to these geometric parameters, an approximate structure of silica aerogel with the density of 0.098 (ρ=0.098) is modeled, whose mechanical properties are subsequently simulated by the finite volume method (FVM). The deformation mechanism and several pure materials parameters can be extracted from the uniaxial compression results, which are used to model the aerogel structure with different geometric properties. In this study, two aerogel models are examined and tuned: one represented with ‘SA-I’ has a Gaussian distribution of pore sizes and ligament diameters; the other represented with ‘SA-II’ has a constant pore size and ligament diameter. In addition, the FVM method is used to simulate and calculate other mechanical properties of these two different models, in which the compression behavior, elastic modulus, and yield stress can be accessed. The computational data are finally compared with experimental results to verify the feasibility of the FVM simulation method developed in this work.

## 2. Experimental Procedure

### 2.1. Preparation

Silica aerogels are prepared using the procedure described by Yang et al. [24], which generally includes two steps: sol-gel synthesis and subsequent supercritical drying process. It is notable that the densities of the silica aerogels can be controlled by adjusting the volume ratio of the starting materials (see Ref. [24] for more details).

### 2.2. Characterization

A JEOL 7000F scanning electron microscope (SEM, JEOL Ltd., Tokyo, Japan) was used to investigate the morphology and microstructure of silica aerogels. The aerogel samples were immersed in liquid N_2_ and then fractured to probe their inner structure. A platinum coating with 5 nm thick was applied before SEM measurements. The relative density of silica aerogels, ρ, was determined using the following equation:

(1) $$\rho =1-\frac{\frac{m_{\mathrm{Gel}}}{V_{\mathrm{Gel}}}}{\rho_{0}}.$$

$m_{\mathrm{Gel}}$ and $V_{\mathrm{Gel}}$ is the mass and the volume of the aerogel, respectively; $\rho_{0}$, the pore volume per unit mass, can be determined by nitrogen adsorption/desorption isotherm data at 77 K (Autosorb-iQASIQ, Quantachrome Instruments, Boynton Beach, FL, USA) using the multi-point Brunauer-Emmett-Teller (BET) method. Mechanical properties of silica aerogel samples were studied using the dynamic mechanical analyzer (DMA, Q800, TA Instruments, New Castle, DE, USA) for compression test at 25 °C. For the compression test, the sample is a cylinder with the height of 1 cm and diameter of 1 cm as well.

## 3. Simulation Method

In the simulation process, a represented volume element with 5 × 5 × 5 unit cells is created and each unit cell size is specifically set without generality restriction. It is notable that this size is selected in order to reduce possible loading introductions along the *z*-axis in the subsequent simulations. As for the modeling of the silica aerogel nanostructures, the SEM and BET method are used to characterize the geometrical properties of the aerogel sample such as pore size, ligament diameter, and particle size. Once these geometric parameters are obtained, the approximate nanostructure of silica aerogel can be reconstructed. Then, the compressive experiment is performed in order to acquire several pure material parameters of the silica aerogel such as elastic modulus, compressive yield stress, etc. Next, these material parameters and the modeled aerogel structure are transferred into the GeoDict software (Ver. 2017, Math2Market GmbH, Kaiserslautern, Rheinland-Pfalz, Germany) [25] in which the mechanical behavior of silica aerogels is simulated by the finite volume method (FVM). The stiffness properties such as Poisson's ratio and elastic modulus can be obtained by calculating linear elastic properties while the strength properties could be studied by computing the nonlinear deformations of lattice structures. As a result, the ElastoDict-VOL module is used to compute stiffness and ElastoDict-LD module is used to calculate strength. It is notable that the periodic boundary conditions are applied to both linear elastic and nonlinear uniaxial compression simulations. In this study, the overall elastic modulus and Poisson’s ratio are obtained under an overall linear compression strain of 5 × 10^−3^. The yield strength is defined as 1% offset stress from the stress–strain curve.

## 4. Results and Discussion

### 4.1. Characterization and Modeling

**Figure 2:**
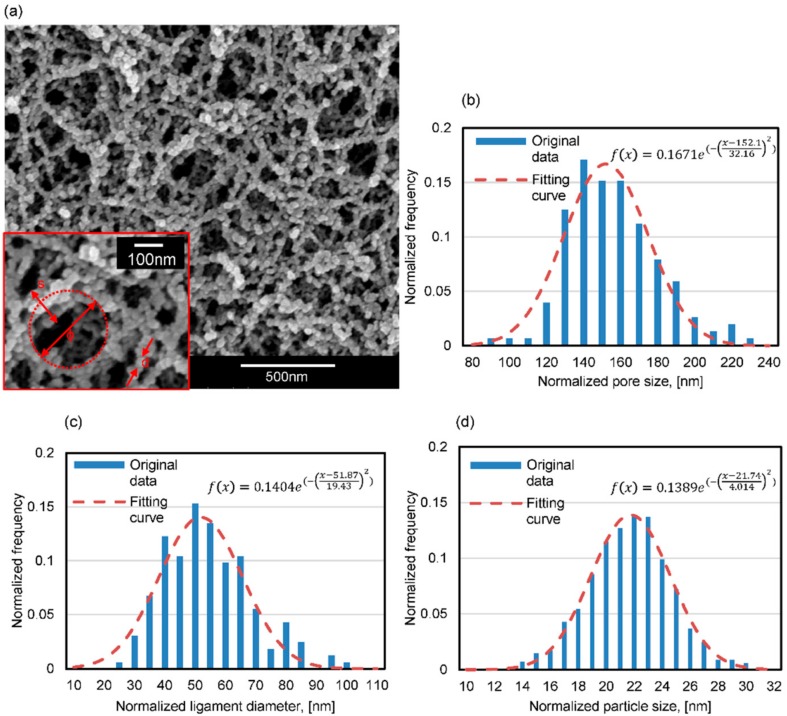
SEM image of the silica aerogel sample with a relative density of 0.098 (**a**), the pore size, ligament diameter and particle size is labelled with *ϕ*, *s* and *d*, respectively; the histogram of distribution of pore size (**b**); ligament diameter (**c**); and particle size (**d**) of the silica aerogel sample. The red dashed line in each panel represents the fitted Gaussian distribution.

Figure 2a presents the SEM image of a silica aerogel sample with a relative density of 0.098. It is observed that a porous, interconnected 3D network structure is obtained by the sol-gel process.

The 3D network frame consists of large number of nanoparticles, which are three dimensionally connected to form a skeletal network and appear as chains. It has been reported that silica aerogels consist of a “pearl-necklace” framework, which is formed by the porous secondary particles [26]. Different geometric parameters of the silica aerogels are also indicated with red rectangular in Figure 2a, such as pore size labeled with ϕ, ligament diameter labeled with s and particle size labeled with d. Specifically, the ligament diameter is defined as the diameter value at the middle of the ligament. As shown in Figure 2a, the ligaments make up the main architecture of the aerogel structure by connecting nodes to other nodes. The ImageJ software (Bethesda, MD, USA) [27] is used to process the SEM images and obtain the statistical results on the geometric properties of the silica aerogel sample. Figure 2b–d show the histogram of the distribution of pore size, ligament parameter, and particle size of the silica aerogel determined by the SEM images. The red dashed line in each panel shows the Gaussian distribution fitting and the fitting function is also indicated in each panel. For example, the mean pore size, ligament diameter and particle size for the aerogel with the relative density of 0.098 is 152.1, 51.87 and 21.74 nm, respectively.

**Figure 3:**
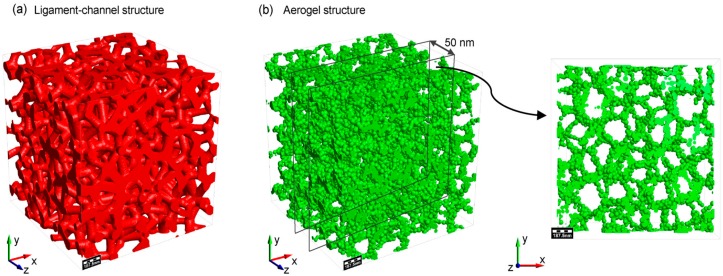
(**a**) the ligament-channel structure reconstructed with the geometric parameters obtained from SEM; (**b**) the overall aerogel structure and its local structure with a size of 250 × 250 × 50.

Figure 3a presents the reconstructed ligament-channel structure based on the geometric parameters of the silica aerogel such as pore size and ligament diameter distribution function in Figure 2b,c, according to the Voronoi tessellation. The ligament is filled up by silica particles with a Gaussian distribution (Figure 2d) to constitute the aerogel structure. The overlap between two adjacent particles is set to 4 nm based on the SEM analysis. Figure 3b demonstrates the overall aerogel structure and its local structure with a size of 250 × 250 × 50.

### 4.2. Compression Behavior

**Figure 4:**
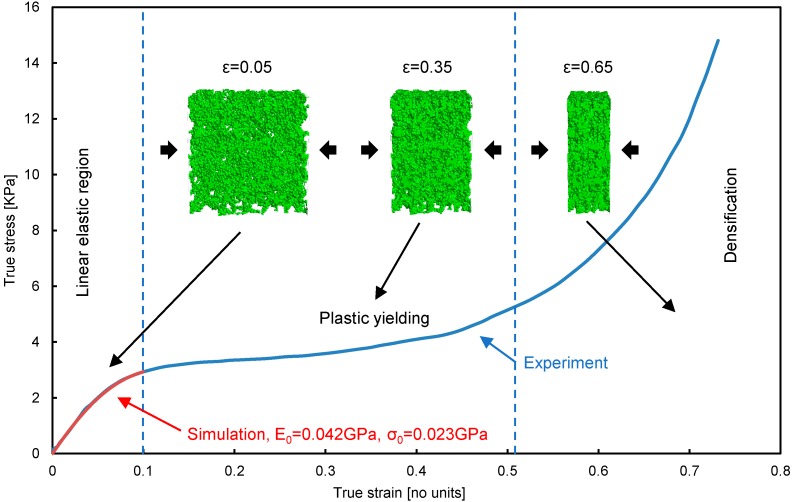
The stress–strain curve of a silica aerogel with relative density of 0.098 under compressive deformation conditions.

In order to gain intrinsic information regarding the mechanical parameters of pure silica materials, such as elastic modulus, yield strength and Poisson’s ratio, the silica aerogel with relative density of 0.098 is firstly examined with uniaxial compression experiment. The compressive experiment shows that the elastic modulus *E*_0_ is 0.042 GPa and the compressive yield stress $\sigma_{0}$ is 0.023 GPa, which is defined as 1% offset stress. The above reconstructed aerogel structure along with the material parameters are imported into the commercial software GeoDict to simulate the compression behavior. Figure 4 shows the stress–strain curve of the silica aerogel sample under compressive deformation conditions from both experiment and simulation, which indicates three deformation stages depending on the compression strain, ε. The first stage corresponds to the stage where the compression strain ε is less than 0.1. It shows a linear elastic region, which is determined by the blending of the structural elements due to the ligaments present in the sample. The elastic modulus, yield strength and Poisson’s ratio can be determined from this stage. In the second stage (0.1<ε<0.5) labeled with ‘plastic yielding region, an evident plateau feature can be observed, which is possibly due to the collapse of the open structures in the sample. The last stage (ε>0.5) is called ‘densification’ stage, where the stress is abruptly increased, which is thought to be the impingement of structural elements against each other and leads to the foam densification. The three inset images correspond to the aerogel with the tensile strain of 5%, 35% and 65% and is used to represent the linear elastic, plastic yielding and densification, respectively. As shown in Figure 4, the silica aerogel sample exhibits similar global compressive deformation behavior as other porous materials [28,29,30]. The FVM method is further used to investigate the microscopic deformation behavior of silica aerogels.

**Figure 5:**
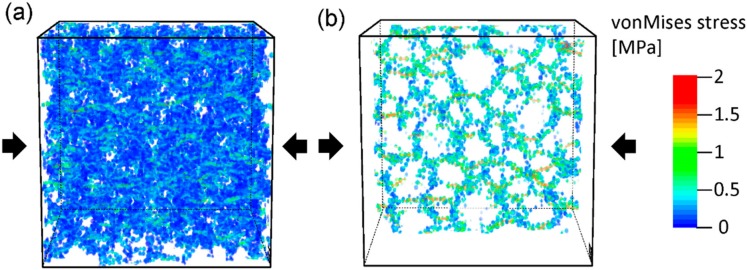
The von Mises stress for the aerogel structure under 0.1 uniaxial compressive deformation. Structural views for the 5 × 5 × 5 (**a**) and its translucent perspectives (**b**) under 0.1 compressive deformation along *z*-axis. The color map indicates the von Mises strain.

Figure 5 presents the von Mises stress for the aerogel structure under 0.1 uniaxial compressive deformation. The structure with 5 × 5 × 5 unit cells (Figure 5a) and its translucent perspective (Figure 5b) indicate that not all ligaments exhibit strain concentration. It can be observed that the main strain distributes in the surface at the junction of node and ligament, which seems to form a train of discontinuous curved surfaces.

### 4.3. Scaling Laws of Elastic Modulus and Yield Stress

The mechanical properties of silica aerogels, particularly the elastic modulus and yield stress, are also of great interest because they provide access to design and develop these aerogels with excellent high strength and stiffness. In this study, two different models are used to simulate the mechanical behavior of silica aerogels: one has a Gaussian distribution of pore sizes and ligament diameters and is represented as ‘SA-I’; the other has a constant pore size and ligament diameter and is represented as ‘SA-II’. However, in both two models, the particle size is assumed to have a Gaussian distribution. Table 1 summarizes the geometric properties of the two kinds of silica aerogels with different relative densities.

**Figure 6:**
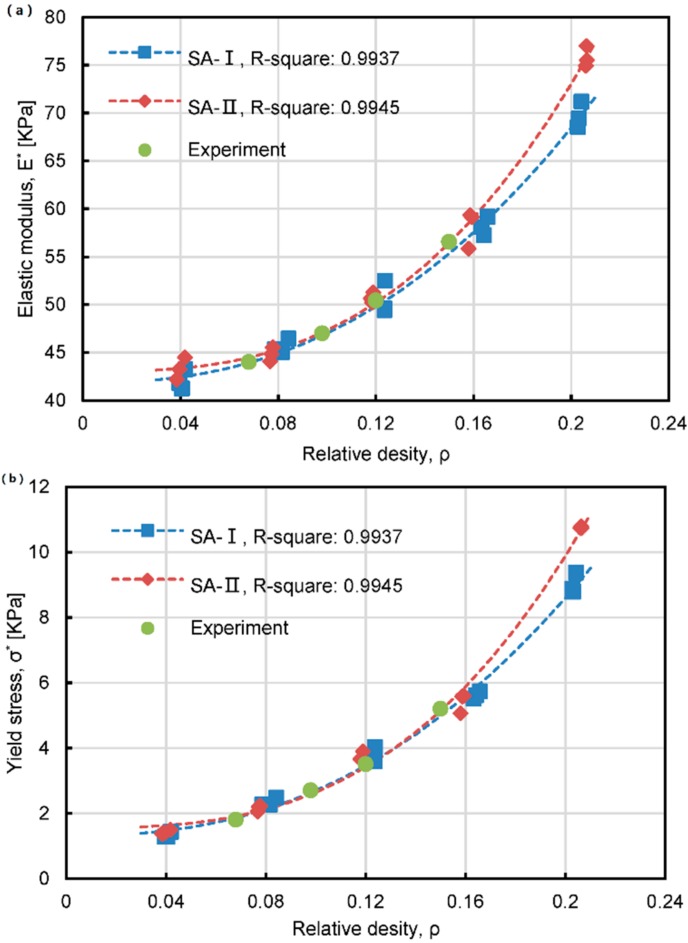
Power law of elastic modulus (**a**) and yield stress (**b**) for the SA-I and SA-II structures. SA-I has a Gaussian distribution of pore sizes and ligament diameters; SA-II has a constant pore size and ligament diameter.

Previous studies have shown that the elastic modulus E and yield stress σ have a power–law dependence on the relative density ρ [31,32]:

(2) $$E\propto \rho^{m}$$

(3) $$\sigma \propto \rho^{n}$$

where m and n both represent constant. Figure 6a,b plots elastic modulus and yield stress for the SA-I and SA-II model with different relative densities, respectively. It can be seen that the experimental data matches well with the simulation curves in both cases. The power law of elastic modulus and yield stress for SA-I and SA-II structures can all be estimated by fitting the data as follows:

(4) $$E_{I}^{*}=1193\rho^{2.363}+41.84,$$

(5) $$E_{\mathrm{II}}^{*}=2723\rho^{2.801}+43.01,$$

(6) $$\sigma_{I}^{*}=342.4\rho^{2.391}+1.307,$$

(7) $$\sigma_{\mathrm{II}}^{*}=933.9\rho^{2.932}+1.551.$$

The power–law index, m, for structure SA-I and SA-II in Equations (4) and (5), is around 2.3 and 2.8, respectively, which is very close to the power–law index in the modulus-density correlation of other silica aerogels [15,33,34,35,36]. As for the power-law index, n, in the strength-density relationship for the SA-I and SA-II structure, it is 2.4 and 2.9 in Equations (6) and (7), respectively, which is also consistent with the literature values. 

Table 2 summarizes some of the published data on the power-law exponent in the strength-density correlation. Woingnier et al. [34] have reported that the power-law exponent is 2.6 ± 0.2 when the density is 0.055–0.5 g cm^−3^ while it is 2.3 ± 0.2 when the density is 0.42–2.2 g cm^−3^. Hence, the simulation results obtained from the FVM method are in very good agreement with experimental and literature results.

As shown in Figure 6, when the relative density is less than 0.08, the values of the elastic modulus and yield strength with SA-II model are larger than SA-I. However, when the relative density is about 0.08–0.12, the elastic modulus and yield strength with model SA-I are very close to or even larger than SA-II. If the relative density is greater than 0.12, the SA-II model again has better stiffness and strength than SA-I. In addition, if the relative density is further increased (i.e., >0.16), the mechanical behavior of SA-II model is even much better than the SA-I one. These results suggest that, when the relative density of silica aerogel is fixed, we can change the pore size and ligament diameter to acquire larger elastic modulus and yield strength. As a result, different strategies can be proposed to optimize the mechanical behavior of silica aerogels. If the relative density is rather too large or too small, the aerogel that has Gaussian distribution of pore size and ligament diameter can be preferentially synthesized in order to acquire structures with higher stiffness and strength. Conversely, if the relative density of the silica aerogel is moderate, such as 0.08–0.12 in this study, the silica aerogel that has fixed ligament diameter and pore size can be preferentially fabricated to achieve better mechanical behavior.

In addition, it should be notable that all the power law relationships in Equations (4)–(7) have intercepts with certain values, which, however, are not predicted in the literature (i.e., Equations (2) and (3)). From Equation (4) to Equation (7), it can be seen that Young’s modulus and yield strength have a certain intercept value even the relative density, ρ, takes a small value. This might suggest that the excellent mechanical performance of silica aerogels can still be maintained even with extremely low densities. Comparing our study with literature results, the most evident difference is that the ligament diameter in our study is constant during the modeling of silica aerogels (see Table 1 for more details). It is known that the tensile properties of porous silica aerogels are largely dependent on the particular orientations among ligaments and the length scale of ligaments, which determine the interconnectivity orders, and thus affect the overall modulus and strength of silica aerogels. For example, Liu et al. [37] once reported that the neck radius in the silica network as well as the strength and stiffness of the particle chains inversely decreases with the increase of the ligament size. Therefore, one possible reason for different fitting curves in our study compared with literature results [31,32] could be due to that the ligament diameter in our case is fixed. As a result, the change of the relative density can only be realized by changing the pore size distribution. This also means that we can control the feature structure of silica aerogels to obtain good mechanical properties (higher stiffness and strength) but maintain a relatively low relative density.

## 5. Conclusions

In this study, the finite volume method (FVM) is used to simulate and calculate the mechanical properties of silica aerogels, which are fabricated with different particle sizes, pore sizes and ligament diameters by the sol-gel method. Two topological aerogel models are first reconstructed based on the geometric parameters of the silica aerogel: one has a Gaussian distribution of pore sizes and ligament diameters; the other has a constant pore size and ligament diameter. Then, the compression behavior of the silica aerogel is simulated using the FVM method, which exhibits a good agreement with the experimental results. The stress–strain curve of the silica aerogel shows the three deformation stages as observed for other porous materials. Through the analysis of the stress–strain curve, the elastic modulus and yield strength of the silica aerogel have been determined to be 0.042 and 0.023 GPa. The mechanical properties of these two models shows a power–law correlation existing between mechanical properties (elastic modulus and yield stress) and relative densities. Furthermore, the power-law indices are consistent with previous studies. These results verify the feasibility of FVM methodology developed in this study. Furthermore, the relationship between relative density and mechanical behavior of two models indicates the influence of pore size and ligament diameter on the elastic modulus and yield stress. These results not only point out future directions of how to optimize the mechanical properties of silica aerogels at a certain relative density, but also indicate that aerogels with ultra-low relative density can also perform high stiffness and strength by controlling the feature structure. Hence, we believe that the FVM simulation methodology developed in this study can be a valuable tool to reinforce and tailor mechanical and even other performance of aerogel and aerogel composites in the future.

## Figures and Tables

**Table 1:** Parameters used in the finite volume method simulation.

	P_LIG_^1^	P_AS_^1^	Pore Size (nm)			Ligament Diameter (nm)			Particle Size (nm)		
			Mean Value	Standard Deviation	Distribution Bound	Mean Value	Standard Deviation	Distribution Bound	Mean Value	Standard Deviation	Distribution Bound
SA-I	0.1	0.04	250	50	100	50	10	20	21.72	2.87	5.74
	0.2	0.08	170	34	68	50	10	20	21.72	2.87	5.74
	0.3	0.12	132	26.4	52.8	50	10	20	21.72	2.87	5.74
	0.4	0.16	112	22.4	44.8	50	10	20	21.72	2.87	5.74
	0.5	0.20	95	19	38	50	10	20	21.72	2.87	5.74
SA-II	0.1	0.04	250	0	0	50	0	0	21.72	2.87	5.74
	0.2	0.08	170	0	0	50	0	0	21.72	2.87	5.74
	0.3	0.12	132	0	0	50	0	0	21.72	2.87	5.74
	0.4	0.16	112	0	0	50	0	0	21.72	2.87	5.74
	0.5	0.20	95	0	0	50	0	0	21.72	2.87	5.74

^1^ Note: P_AS_ indicates the density of the aerogel and P_LIG_ is the density of the ligament. P_AS_ is equal to 0.04 P_LIG_ in the simulation.

**Table 2:** Exponent for the power law relation between yield strength and density of porous silica.

Reference	Power-Law Exponent	Density (g/cm^3^)	Observations
Woignier et al. [34]	2.6 ± 0.2	0.055–0.5	Experiment
Woignier et al. [34]	2.3 ± 0.2	0.42–2.2	Experiment
Murillo et al. [15]	2.25–3.14	0.05–0.5	Atomistic model
